# Brain Network Evolution after Stroke Based on Computational Experiments

**DOI:** 10.1371/journal.pone.0082845

**Published:** 2013-12-20

**Authors:** Wei Li, Yue Huang, Yapeng Li, Xi Chen

**Affiliations:** 1 Image Processing and Intelligent Control Key Laboratory of Education Ministry of China, Wuhan, P. R. China; 2 Department of Intelligent Science and Technology, College of Automation, Huazhong University of Science and Technology, Wuhan, P. R. China; 3 Department of Systems Science and Engineering, College of Automation, Huazhong University of Science and Technology, Wuhan, P. R. China; Cuban Neuroscience Center, Cuba

## Abstract

Stroke is a frequently-occurring disease threatening the human nervous system. As a serious debilitation affecting a large-scale, hierarchical, and vastly complex electrochemical system, stroke remains relatively misunderstood. Rehabilitation mechanisms and means have suffered from this lack of systematic understanding. Here we propose an evolution model to simulate the dynamic actual evolvement process of functional brain networks computationally in an effort to address current shortcomings in the state of the field. According to simulation results, we conclude that the brain networks of patients following acute stroke were characterized by lower small worldness and lower quantity of long-distance connections compared with the healthy condition. Moreover, distance penalization may be used to describe the general mechanism of brain network evolution in the acute period after stroke.

## Introduction

Stroke is a common, debilitating disease threatening the human nervous system. Among 731,000 incidents of stroke and 4 million stroke survivors recorded in the United States annually [1], 25% of patients return to a level of daily physical function [2]. Indeed, the process and mechanism of nervous system rehabilitation after stroke are still not very clear. Addressing this issue and its complications has direct benefits for the state of patient care and recovery.

Neuroimaging technology has been widely used to investigate changes in nervous system function after brain injury. Such studies indicate that the functions of the nervous system change greatly in both the acute period and chronic rehabilitation process after stroke [3]–[5], and also show that after a subcortical stroke brain activity changes in most of related cortical regions rather than the perilesional area [6], [7].

Recently, complex network theory has been applied to neuroscience research and has produced meaningful results [8]. Graph theory is a natural framework for the mathematical representation of complex networks. According to this theory, the brain can be described as a graph consisting both of nodes representing regions or voxels as well as connections representing structural or functional connectivity between nodes [9]. Meanwhile, other studies have demonstrated that brain activation changes are closely related to brain functional connectivity resulting from changes in neural pathways between cortical regions of the brain after neural injury [10], [11]. Carter et al. suggested that cortical activation after stroke could be assessed with greater accuracy at the level of an entire brain network rather than just through qualitative analysis limited to the site of structural damage [12]. Furthermore, recent studies [13]–[15] indicate that the function of any brain regions must be resolved in conjunction with other brain regions (the ‘network’) with which it interacts both while at rest and during active behavior. Some of these implications were forecasted many years ago by early neurologists such as Jackson, Andral, Prince, von Monakoff, and Head [16], who proposed that neurological deficits do not simply reflect the primary effect of a lesion but also the secondary effects of the lesion on other structures. Using resting fMRI data from 3 week-old to 2 year-old neonates, Gao et al. found that the brain possessed small-world topology features immediately after birth, characterized by a remarkable improvement in whole brain wiring efficiency in 1 year olds gaining stability in 2 years olds [17]. Although graph theory has attracted considerable attention in brain network research [18], [19], it has seldom been applied to the study of changes in brain connectivity following stroke [20]. In one study, a motor execution network was found to gradually shift towards a random mode during the recovery process after stroke, which suggested a less optimal reorganization of functional rehabilitation of affected limbs [21]. This study compares the topological profiles of patients and controls, simulating the dynamic evolution process of brain network after stroke.

Network evolution, as a new approach developed in complex network theory, has previously been shown to be an effective method for simulating the dynamic changing process of complex networks. Gross used network evolution principles to explore the formation process of networks based on the network formation mechanism [22]. A large number of studies in network evolution of real systems have been published [23]–[27], and the concept of network evolution introduced by De Vico Fallani [28] shows that the brain network approaches an optimal structure [29] and that the coupling strength is inversely proportional to the distance between nodes [30]. Vertes et al. used brain anatomy distance and node degree as parameters to calculate the connection probability between nodes in brain networks, and then established a hemisphere-brain network from isolated nodes through simulation from scratch, showing that the evolved brain network is similar to the real human brain network at a macro level [31]. While current brain network evolution studies consider connection probability, it has been demonstrated that couplings or connections between regions in brain or nodes in brain network are not fixed, but change dynamically as a result of aging or disease [32], [33] ie., they possess a high disconnection probability. Our study attempts to consider the occurrence of disconnection besides connection and simultaneously emphasize the impact of haphazard factors during the evolution process of brain networks.

In this paper, we establish a computational experiment platform utilizing brain network evolution to simulate the dynamic actual evolvement process of functional brain networks after stroke. Firstly, using task-based fMRI data, the functional brain networks of controls and patients following acute stroke were characterized by graph theory respectively. By comparing the topological parameters of the brain network between the two groups, common features of topological alteration after stroke were extracted, and the evolution rules and strategies in accordance with healthy persons and patients in the acute period were established. The connection probability and disconnection probability were both considered in the evolution rules to simulate nodal coupling and decoupling in brain network evolvement processes after stroke.

## Materials and Methods

### 1. Participant

Five right-handed patients (4 male and 1 female; mean age 52.4 years; range 31–65 years) with stroke were enrolled from the inpatient services at the Tongji Hospital of Huazhong University of Science and Technology (Wuhan, P.R.China). Five right-handed healthy controls (4 male and 1 female; mean age 49.4 years; range 30–62 years) were also recruited. There were no group-differences in understanding or education background. The fMRI data of all patients were acquired when they were just admitted to hospital. All subjects gave their written informed consent and the study protocol was approved by the Ethics Committee of Tongji Medical College, Huazhong University of Science and Technology.

All patients exhibited the following inclusion criteria: 1) first occurrence of ischemic stroke; 2) study participation within two weeks after stroke; 3) motor deficiency with acute unilateral loss of hand strength (Grade
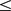
4 with the Medical Research Council (MRC) scale (0–5, 5 = Normal)); 4) regular motor function of the ipsilateral hand. Exclusion criteria were as follows: 1) language or cognitive deficits which would impact cooperation in fMRI examination; 2) significant somatosensory (light touch or proprioception) deficits of the stroke-affected hand; 3) mirror movements; and 4) contraindication to magnetic resonance imaging.

**Figure 1:**
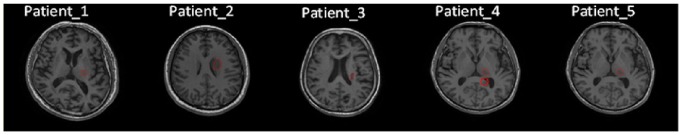
Illustration of lesion location in red for each patient.

The clinical characteristics and the medication of the patients are summarized in Table 1. All five patients had local areas of infarction on the left side of brain and had contralateral motor deficit (Table 1). The illustration of lesion location is shown in red in Figure 1.

**Table 1:** Clinical and demographic data.

Patientnumber	Age	Sex	Affectedhand	BarthelIndex	Fugl-MeyerAssessment ofMotor Function	NIHSS	Localization of infarct	Medication
1	65	M	Right	80	69	3	Left thalamus	aspirin and atorvastatin
2	46	M	Right	60	63	2	Left caudate nucleus	aspirin and atorvastatin
3	60	F	Right	65	78	1	Left paraventricular corona radiata	aspirin and atorvastatin
4	31	M	Right	75	86	1	Left thalamus and corpus callosum	aspirin and atorvastatin
5	60	M	Right	55	53	3	Left thalamus	aspirin and atorvastatin

### 2. Experimental Paradigm

**Figure 2:**
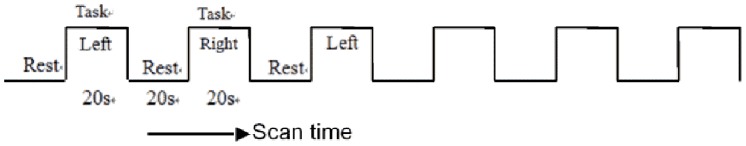
Paradigm design.

All the subjects were instructed to execute alternating unilateral finger-to-thumb opposition movements at a frequency of 1 Hz in a block-design fMRI paradigm. The task occurred in 20-s blocks of movements alternated with 20-s intervals rest periods. The whole fMRI procedure lasted for 260 s as shown in Figure 2. During fMRI procedure, the subjects kept their eyes open and their head motionless. The motor task was performed by subjects following visual cues. Prior to scanning, the subjects were instructed and trained in the task in the same way until they understood the task and were able to adequately follow visual cues and instructions. The performance of the motor task was monitored by a doctor in the inspection room.

### 3. Data Acquisition

MRI scans were acquired on a 3T GE Signaxs scanner (General Electric) with a custom-built head coil. A high resolution T1-weighted SPGR (spoiled grass gradient recalled) inversion recovery 3D MRI sequence was performed for each subject with the following parameters (TI = 400 msec; TR = 6.5 ms; TE = 2.1 ms; Flip Angle = 15 degrees; FOV = 25.6 cm; 132 slices in coronal plane; 256×256 matrix; 1NEX, Acquired Resolution = 1×1×1.1 mm).

Blood oxygenation level dependent (BOLD) signal was collected with a T2-weighted gradient echo spiral in-out pulse sequence [34] with the following parameters (TR = 2,000 ms, TE = 30 ms; Flip Angle = 90 degrees; 1 Interleave; FOV = 24 cm; 64×64 matrix). A total of 32 axial slices (5.0 mm thickness, 0 mm skip) parallel to the AC-PC line with whole brain coverage were obtained with a temporal resolution of 2 s. 120 images were obtained from a task lasting 4 min. Structural and functional scans were acquired in each scan session.

### 4. Preprocessing of Functional MRI Data

The functional MRI data set was preprocessed using SPM8. First, the dicom data set was converted into a *.img/*.hdr document. Then, slice timing was used to correct for time-domain. Afterwards, all image volumes were realigned to the mean volume. Subjects whose head displacement were more than 2 mm in x,y,z direction or whose head rotation exceeded 1° were excluded (2 patients were excluded under this criteria and are not shown in Table 1). Using the unified segmentation approach [35], function images were normalized to the MNI template (voxel 3×3×3 mm). In order to decrease spatial noise, volumes were smoothed by a 6-mmfull-width half maximum Gaussian kernel. Finally, datasets were drifted and filtered with 0.01 Hz–0.08 Hz. Covariates were removed after preprocessing.

### 5. Construction of Functional Brain Network

Pearson correlation between BOLD time courses of brain regions were used to construct functional brain networks. The effect of covariance was eliminated from the image after pretreatment by de-noising, and each brain was divided into 90 regions according to the AAL template. Then Pearson correlation was calculated between BOLD time courses of any pair of regions. High negative correlation is also regarded as a close relation between brain regions. The weights in adjacency matrixes were absolute values of correlation between brain regions. A 90*90 adjacency matrix was acquired, and each unit was assigned a value from 0 to 1. Bullmore has shown that each region conforms to the profile of a realistic brain model when the density of functional brain network varies between 8% and 16% [36], [37]. Hence, strongest links were preserved to construct functional brain networks with densities ranging from 8% to 16% for subsequent analysis.

Graph theory is the natural framework for the exact mathematical treatment of complex networks. Thus, the functional brain network was described by a graph in this study. A graph consists of a set of vertices (or nodes) and a set of edges (or connections) indicating the presence of some sort of interaction between the vertices. The adjacency matrix *A* contains the information about the connectivity structure of the graph. When a link connects two nodes *i* and *j*, the corresponding entry of the adjacency matrix is 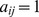; otherwise 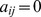.

### 6. Small-World Network

Watts and Strogatz [38] have shown that well-ordered networks are characterized by a high cluster index *C* and a short characteristic path length *L*. Such near optimal models are designated as “small-world” networks. When the threshold is exceedingly high, some nodes may become disconnected from the graph which poses problems with regards to the computation of *C* and *L*. Latora and Marchiori [39] have proposed the concept of network efficiency to address this problem. The clustering coefficient *C* and characteristic path length *L* of unweighted networks conceptually correspond to the local and global efficiency of weighted networks, respectively. The efficiency of the path between two nodes is the inverse of the shortest length (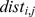) between the nodes. In cases where a path does not exist, the length is considered to be infinite, and the efficiency is zero. The average of all pair-wise efficiencies is the global efficiency of the graph:
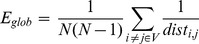
(1)


Furthermore, the local efficiency for each node can calculated as the global efficiency of the neighborhood subgraph 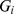 of the node. The local efficiencies across all nodes within the network are further averaged to estimate the network local efficiency 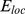 as follows:
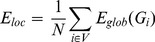
(2)


In terms of network efficiency, a small world network is the one with high 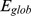 and 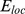 (i.e., very efficient both in global and local information transfer) [40]. So the small worldness (SW) is then defined [41] as:
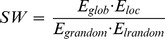
(3)



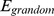
 and 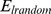 denote the global efficiency and local efficiency averaged over surrogate random networks, where each edge was randomly rewired in a graph.

### 7. Evolution Model

In order to simulate the dynamic evolvement process of the brain nervous system in the acute period, an evolution model was presented as follows:
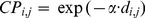
(4)


Here, 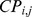 is used to control the occurrence of connection and disconnection between the node 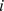 and the node 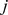, 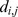 is the anatomical distance between the node 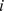 and the node 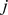, 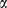 is the penalty parameter of the connection distance.

In this model, the occurrence probability of connection between nodes is inversely proportional to the anatomical distance between nodes. Constrastingly, the occurrence probability of disconnection of an edge between two nodes is proportional to the anatomical distance between nodes.

A random function was applied to imitate the influences of haphazard factors to connection and disconnection. If Eq.5 is satisfiable and there is no edge between nodes 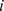 and 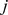, then a link between the nodes 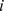 and 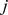 will be established. And if Eq.6 is satisfiable and there is just an edge between nodes 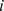 and 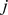, then the link between the nodes 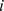 and 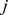 will break. The indexes 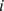 and 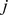 are selected randomly and repeatedly to ensure the evolvement process is rational. Following this approach, the larger 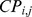, the greater the probability of establishing a link between nodes 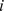 and 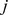.
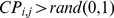
(5)

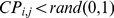
(6)


Here the function 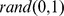 picks a random number in (0, 1) that is in the interval from zero exclusive to one exclusive.

To find an appropriate value of 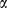 which best fits the data, we applied a differential evolution algorithm (DE) to a cost function (Eq.7) based on the P value for the difference in distance distribution between a set of simulated networks and the data derived from patients. A differential evolution algorithm (DE) is a stochastic direct search method to handle such problems with nonlinear cost function [42].
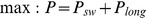
(7)


**Figure 3:**
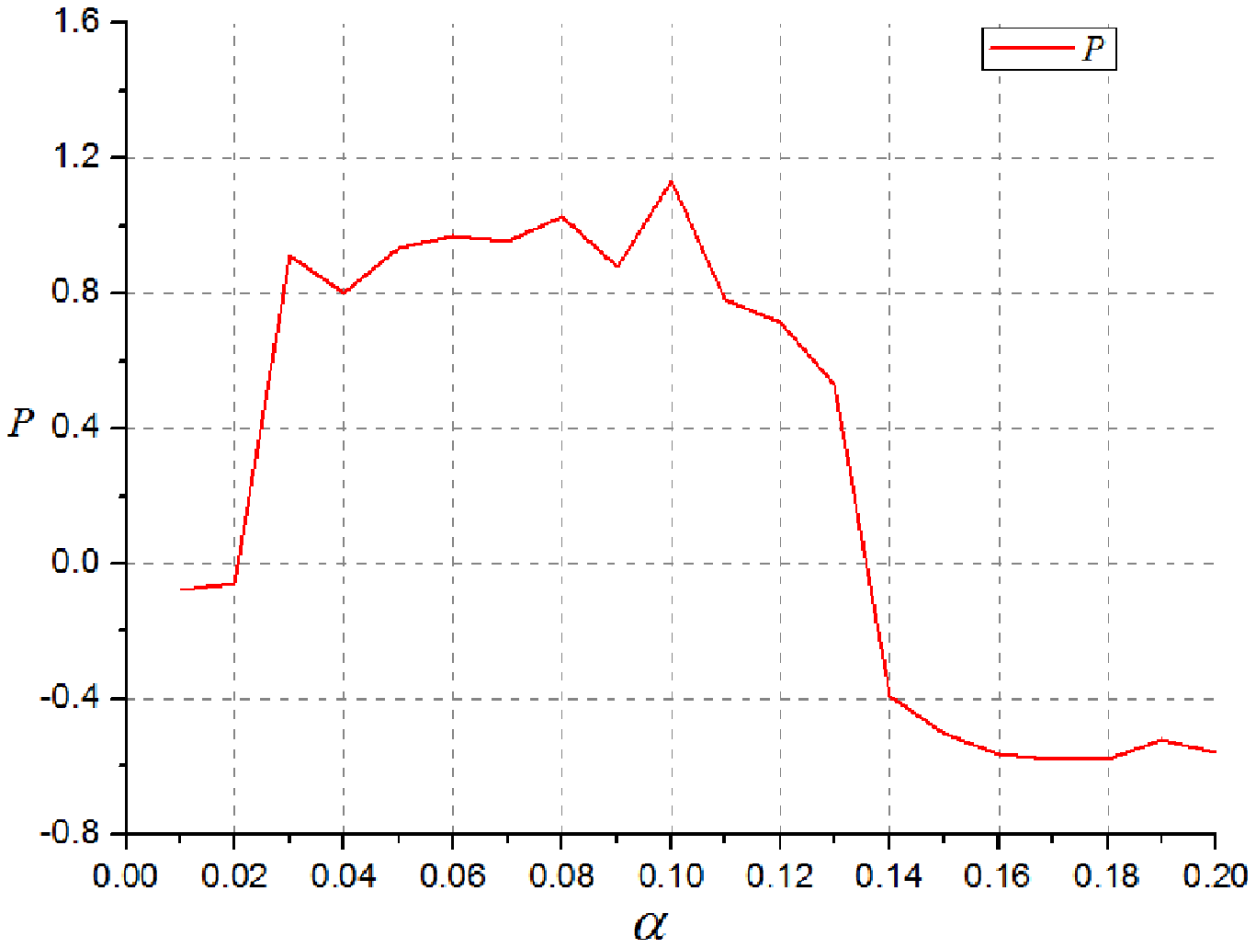
P values of simulated networks obtained by varying each parameter. P with higher value means the feature of simulated networks is more similar to that of brain networks derived from patients.

Here, 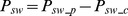 and 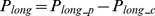. 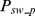 and 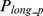 are P values of Wilcoxon rank tests comparing the small worldness and the number of long-distance connection between simulated networks and brain networks of patient group. 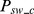 and 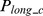 are P values of Wilcoxon rank tests comparing the small worldness and the number of long-distance connection between simulated networks and brain networks of control group. P values of the networks were obtained by varying each parameter around the optimal value (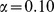) (Fig. 3).

### 8. Computational Experiments of Network Evolution

Studies of brain injury and their impacts remain extremely difficult due to the following two facts: 1) trauma cannot be reduced to simple cases and must be integrated as a whole, and 2) situations or events are not repeatable or re-constructible such that discrete experiments are virtually identical. While traditional approaches are not effective for addressing these problems, new concepts and methods developed in complex systems may provide a potential solution [10]–[12], [17], [43]. This consideration is the motivation for establishing a computational platform to simulate the dynamic evolvement process of functional brain network after brain injury based on a newly developed computational theory of complex systems using computational experiments.

Computational experiments method was firstly proposed by Bankes in 1993 to investigate highly complex systems. He implied that it was difficult to construct a deterministic model to accurately simulate a highly complex system consisting of numerous self-adaptive agents. The computational experiments method provided a solution for such problems in the form of a computation platform [44]. This is a natural extension of computer simulations in the sense that accuracy to real systems is no longer the only criterion for model construction. Instead, a “model” can be considered as an alternative to the reality and, for experimental purposes, the “equivalent” of a real system.

In our study, the initial phase of the evolution is the brain network of a healthy control, and the evolution ends when the difference between the simulated network and brain networks of patients following stroke is at a minimum. According to this procedure, the simulated evolution can be implemented repeatedly until a brain networks corresponding to patient controls reappear. Multiple evolutions yield multiple brain-like networks, and are limited in the sense that individual brain-like networks do not necessarily correspond with the discrete outcomes inherent in a small patient set.

## Results

### 1. The Comparison between Patients and Controls

**Figure 4:**
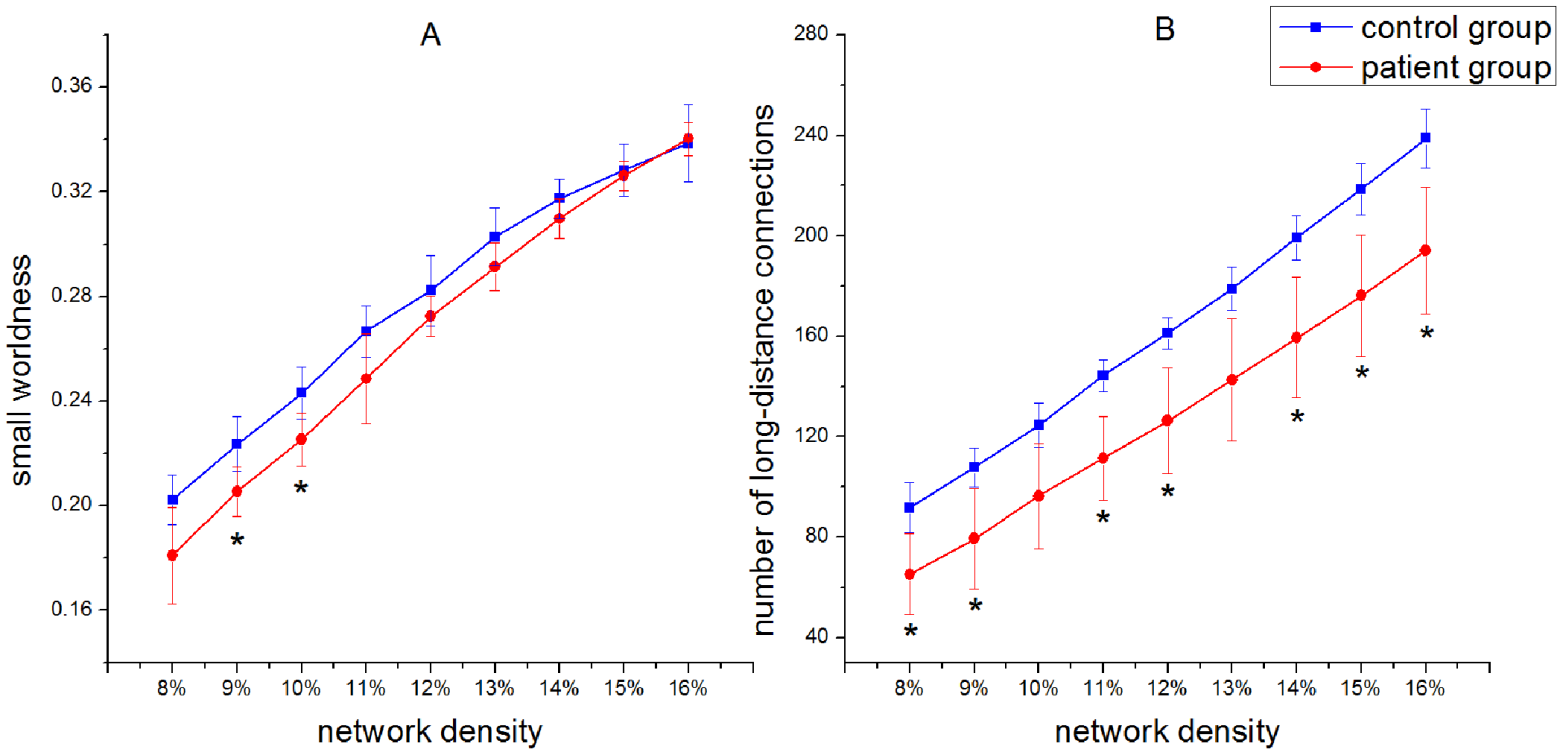
The small worldness (A) and the number of long-distance connections (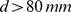) (B) of brain networks in the control group and the patient group. Vertical lines denote the standard deviation of each group. Asterisks denote significant differences (Wilcoxon rank test, 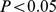) between two groups at corresponding network densities.

**Figure 5:**
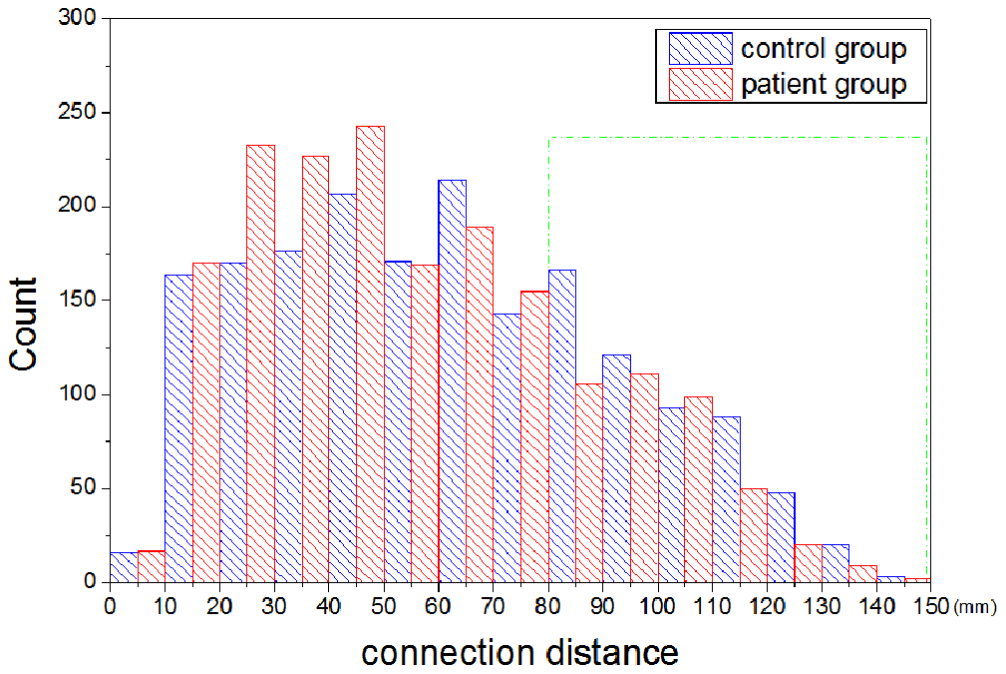
Comparison of the connection distance between controls and patients. The distance of connections in brain networks of control group (blue) and patient group (red) were shown.

To evaluate simulation accuracy, we quantified the changes which occurred in the topological profile of the patient group relative to the control group. We found that the small worldness of a brain network in the control group is greater than that of one in the patient group (Fig. 4A), and that the difference is significant when the density of brain network is between 9% and 10%. We observed that the number of long-distance connections (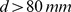) of the patients decreased, relative to the controls (Fig. 4B). Only in the case of network density equalling 9% do both the small worldness and the number of long-distance connections show a statistical difference between patients and controls. A brain network of 9% density was therefore chosen for brain network evolution. In Fig. 5, a histogram was used to graphically summarize and display the distribution of the connection distance of brain networks of 9% density. The distance between connections of brain networks derived from all subjects in the control group and the patient group was calculated. A Kolmogorov-Smirnov test was also used to compare the probability distribution for connection distance between brain networks of control group and patient group. The P value from the Kolmogorov-Smirnov test is very small (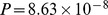), which shows that the probability distributions of controls and patients cannot be drawn from the same contiguous population.

### 2. Simulation Results

The evolution model was used to simulate the evolution process of brain network in the acute period, defined as from pre-stroke to two weeks after stroke. The evolution of each control subject was simulated 20 times to obtain 20 networks, resulting in 100 outcomes.

**Figure 6:**
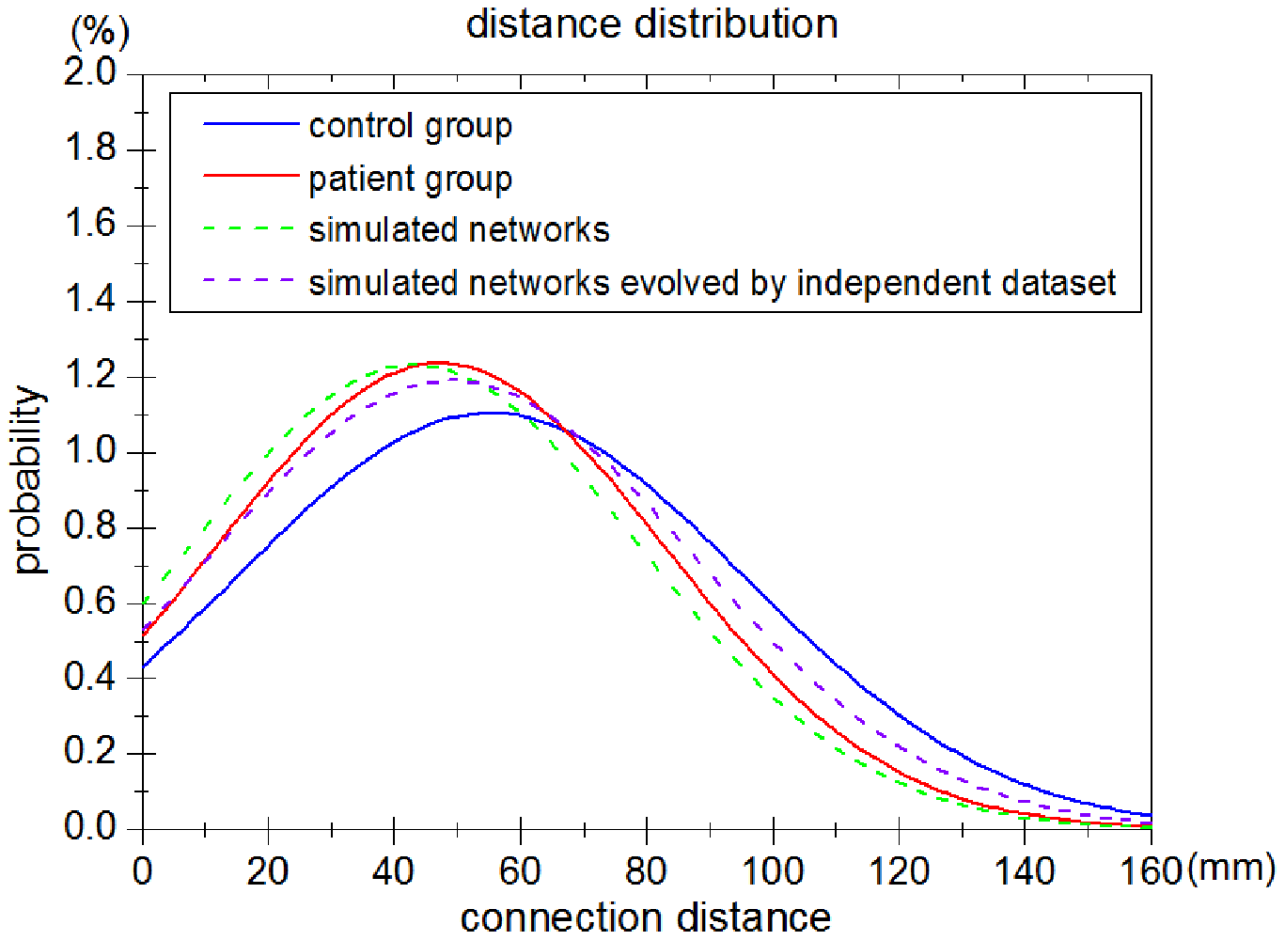
Comparison of distance distribution between networks simulated by the evolution model and brain networks derived from experimental fMRI data from the patient group of 5 subjects. The distance distribution of the overall connections in the brain networks of the control group (blue line) and the patient group (red line) are shown in the graph. The dashed green line shows the distance distribution of the overall connections in networks simulated by the evolution model. The dashed purple line shows the distance distribution of the simulated networks evolved by a second independent dataset.

Firstly, we measured the distance distribution (the probability distribution of Euclidean distance between connected pairs of regions) of a set of simulated networks and brain networks derived from experimental fMRI data (Fig. 6). The distance distribution of simulated networks captured by the evolution model was found to approach that of the patient group. The probability distribution curve of the connection distance of simulated networks was almost equal to that of brain networks of patients following stroke. The results of the Kolmogorov-Smirnov test show that the two independent distance distributions of patient group and simulated networks are drawn from the same underlying continuous population. Additionally, the Kolmogorov-Smirnov test was performed between the simulated brain networks and the control brain networks. The results indicate that the distance distributions of the control group and simulated networks are not drawn from the same population.

**Figure 7:**
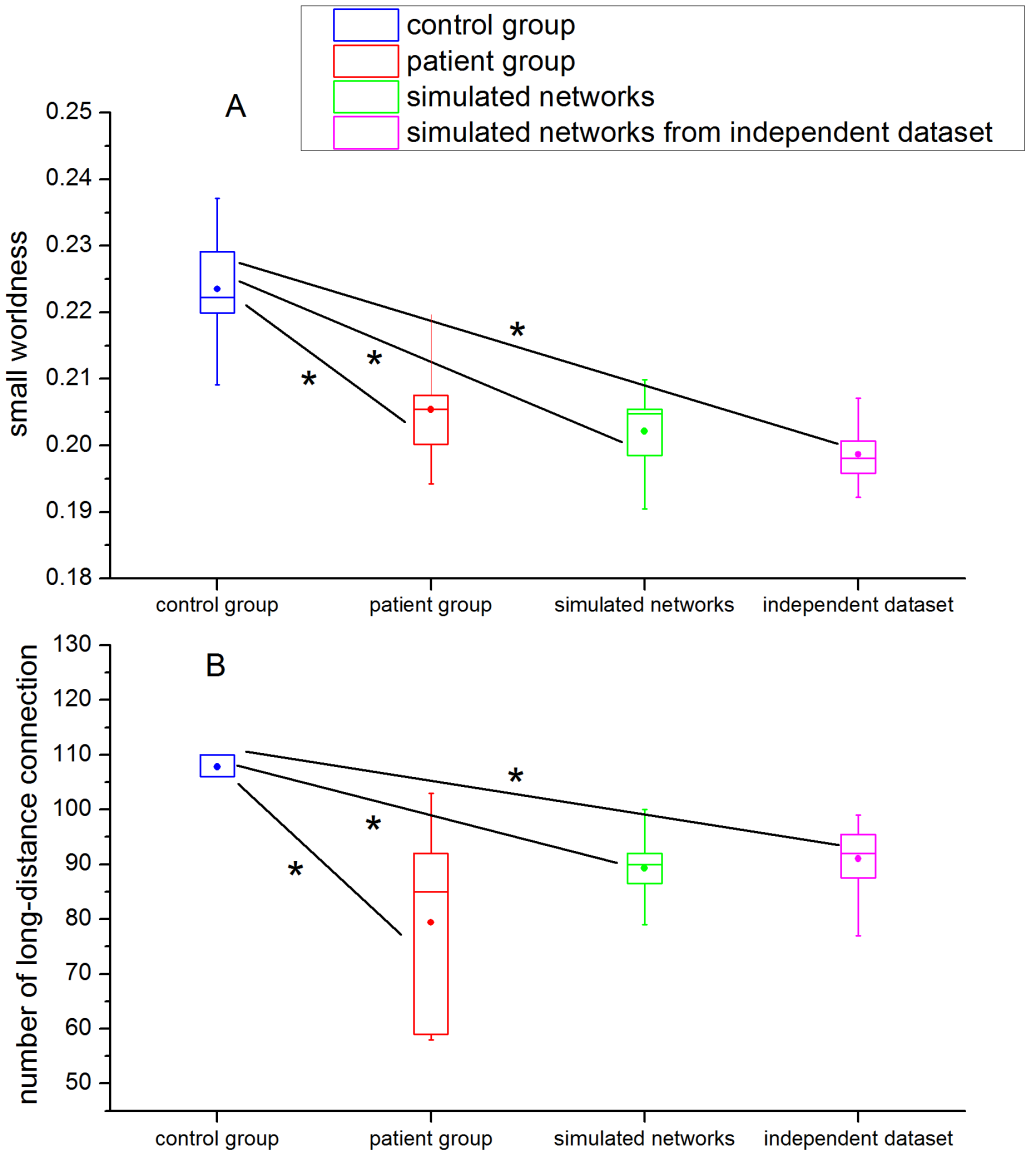
Mean values of the small worldness (A) estimated from the experimental fMRI data and the simulated networks. Rectangular boxes indicate the standard deviations and dots indicate the mean values. Asterisks denote significant (Wilcoxon rank test, 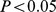) difference between conditions. The same applies for rectangular boxes, dots and asterisks in panel B. Mean values of the number of long-distance connection (B) estimated from the experimental fMRI data and the simulated networks.

**Figure 8:**
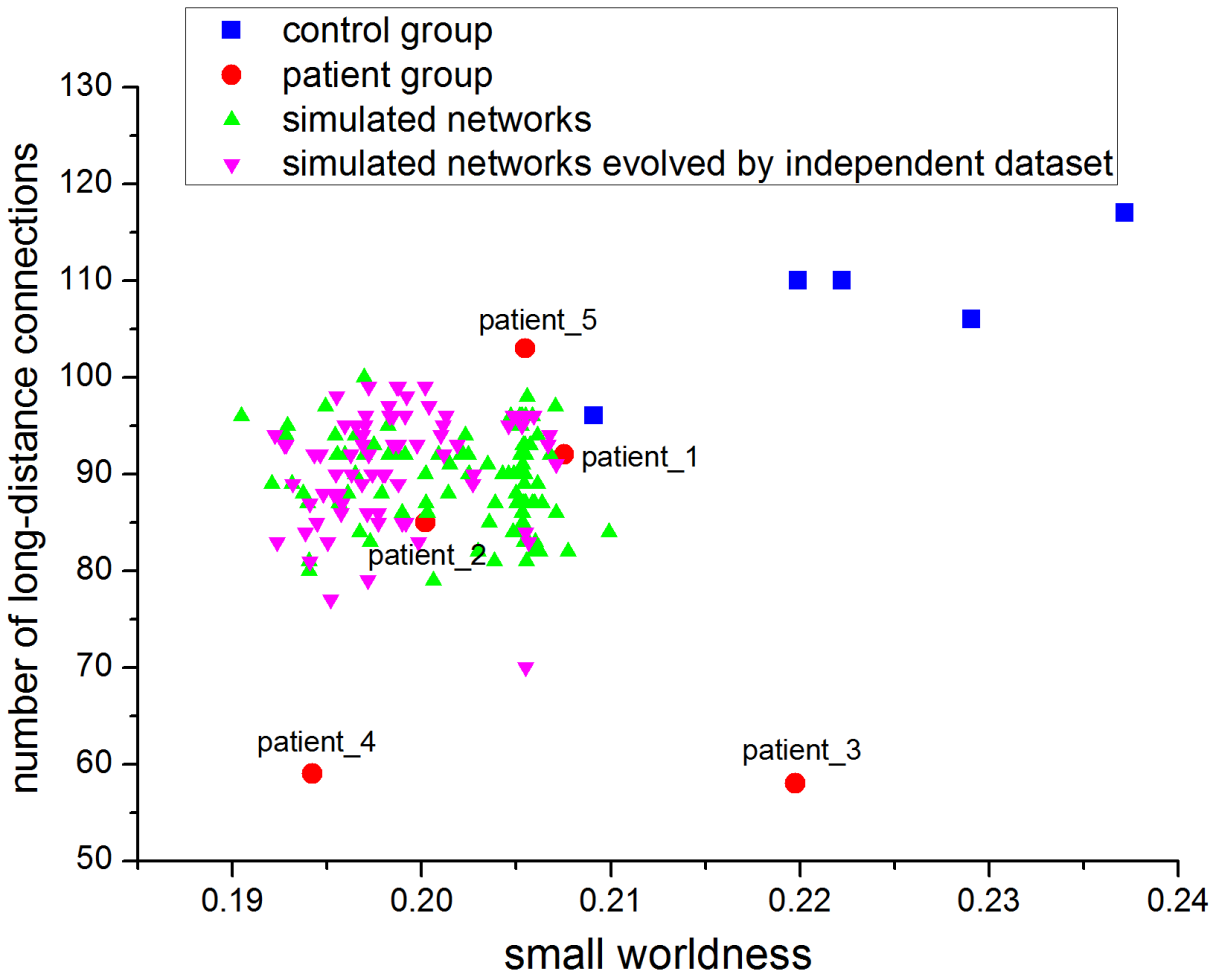
Scatter plots of the small worldness and the number of long-distance connections. X-axis denotes the small worldness; Y-axis denotes the number of long-distance connections (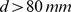). All the values are grouped by control group or patient group while the green triangles represent the distribution of all simulated networks. The purple triangles represent the simulated networks evolved by a second independent dataset. All patients are labeled in line with the patient numbers in Table 1.

Secondly, the small worldness and the number of long-distance connection were compared between two groups (patient and control) and the simulated networks (Fig. 7). The small worldness was significantly lower in patient group (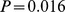) and simulated networks (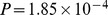) than in control group (Fig. 7A). The number of long-distance connections has the same property as the SW index (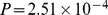 for patient group; 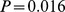 for simulated networks) (Fig. 7B). Figure 8 simultaneously displays the small worldness and the number of long-distance connections in the two groups (patient and control) studied with those obtained from the simulated networks having the same number of nodes and links. It was found that all scatters representing simulated networks are distributed in the region which corresponds with the features of patients, that is, the lower small worldness and the lower quantity of long-distance connections (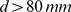) with respect to control group (Fig. 7 and Fig. 8).

Finally, we used an independent fMRI dataset (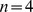 healthy controls) to simulate the evolution process after stroke using our evolution model. In this way we tested the appropriateness of the fit of the model parameter on an independent set of experimental data that had not been used for model estimation. The evolution model generated a good approximation between the simulated networks evolved by the independent fMRI dataset and brain networks of patients (Fig. 6, Fig. 7, Fig. 8).

## Discussion

### 1. The Comparison of the Functional Brain Network between Controls and Strokes

In a functional brain network, varying topological properties are due to the ocurrence of differing arrangements of connections between brain regions after onset of stroke, although the number of connections before and after stroke remains the same in both healthy subjects and patients. The arrangement of functional links between different brain sites can affect the level of information processing and signal synchronization, and have a large influence on experimental results.

Small worldness is an index of small world structure, reflecting an optimal structure associated with rapid synchronization and information transfer, minimal wiring costs, as well as a balance between local processing and global integration. Our results suggest that small worldness significantly decreases after stroke (Fig. 4A). This finding has been described in previous studies. De Vico Fallani et al. proved that small worldness decreases significantly in evolution networks of patients compared with controls, reflecting a lower capacity among patients for information transfer between distant brain regions after stroke [45]. A recent study of his group found that patients with subacute stroke have significantly lower small worldness of the affected hand when compared with the unaffected hand during the motor imagery [46]. And Tsirka demonstrated that patients with brain injury have sub-optimal network organization, as reflected by a decrease in small worldness value [47]. Therefore, small worldness is a useful index to evaluate brain function.

We also found that the number of long-distance connections (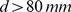) decreased after stroke (Fig. 4B). Stam [8] hypothesized that the topological structure of functional networks is probably restrained by anatomical factors. Furthermore, Alexander-Bloch verified this forecast by comparing the connection distance in functional brain network between healthy controls and patients with childhood-onset schizophrenia. He argues that topological disturbances of functional network organization can be caused by excessive “pruning” of short-distance functional connections in schizophrenia [30]. Thus, the significant decrease in small worldness after stroke may arise from reduction of long-distance connections in patients (Fig. 7).

### 2. The Evolution Model

Our evolution model emphasizes a property of the brain network: the anatomical distance between nodes. According to our evolution rules, the attributes of all vertices and edges are modifiable, implying that all the regions of cortex are probably impacted after stroke. These evolution rules are consistent with some results of previous studies using models to simulate the dynamic effects of brain injuries. Honey and Sporns implemented two models of oscillatory cortical interactions and found that lesion effects extend beyond the immediate neighbors of the lesioned site, and that the amplitude and dispersal of nonlocal effects are influenced by cluster patterns in the network [48]. Alstott et al. adopted a computational model to simulate the dynamic effects of lesions placed in different regions of the cerebral cortex, and found that lesions produce specific patterns of altered functional connectivity among distant regions of cortex, supporting the claim that lesions affect both cortical hemispheres [49].

It has been reported that small world structure is one of the most important features of the human brain, which has been shown to pursue a balance between local processing and global integration with information transfer at a minimal energy cost [47], [50], [51]. In mammalian brain networks, it has been shown that energy consumption is proportional to the physical distance between brain regions in information transfer [52]. Castellanos et al. reported that the energetic cost is one of the most altered topological parameters after brain injury [53], implying that energy consumption related to information processing in a brain network changes greatly after brain injury. Therefore, the Euclidean distance of connections in the brain network is useful for showing the alteration of energy consumption during post-stroke brain network evolution.

In our study, the concept of disconnection was emphasized and probabilistic factors were introduced to the simulated evolution process. If connection takes place in the evolution process solely without disconnection, the network density will necessarily increase on account of the increase on edges. Moreover, Gong and Wang demonstrated that the interactions or connections between nodes in a brain network are not invariable, but change dynamically with age [32], [33]. Links with high connection probability 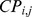 between nodes 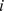 and 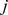 are likely but not guaranteed to be established. The same situation arises for the condition of disconnection. According to figure 8, the results simulated from each control are very similar, but not exactly uniform. We considered that this might be due to a small group size in this study.

### 3. The Evaluation of the Evolution Networks

The nervous system is a large-scale, hierarchical and self-adapting complex system [54], which consists of vast numbers of neurons connected closely through electrochemical action. Deterministic and probabilistic factors coexist in the plastic process of the nervous system. This duality is a function of uncertainty in the evolution of brain networks. The computational experiments method is therefore an effective approach to explore this complex system. Using the computational experiments method, we have simulated the evolution process of brain networks repeatedly impacted by deterministic and probabilistic factors, and obtained experimental results of brain network evolution with not only common features but also intrinsic uncertainties aroused by probabilistic factors (Fig. 8).

The networks simulated by the evolution model confirmed our hypothesis that a decrease in small worldness can arise from the reduction of long-distance connections in the networks of patients following stroke (Fig. 7). Simulation results show that the distance distribution of evolution networks evolved from the brain networks of controls is very similar to the distance distributions of brain networks of patients (Fig. 6). Meanwhile, a decrease in small worldness is associated with a reduction of long-distance connections in brain-like networks (Fig. 7). Scatters that represent evolution networks are distributed in the region which corresponds with the features of patients (Fig. 8). According to our simulation results, we conclude that the brain networks of patients following acute stroke were characterized by lower small worldness and lower quantity of long-distance connections compared with the healthy condition. Moreover, our evolution model can succesfully simulate the dynamic brain network process of post-stroke patients in the acute period.

### 4. The Methodology Issues

The connection distance, the primary parameter of the connection probability function and the disconnection probability function, is a rough measure representing the information transmission distance between brain regions. In practice, various brain regions are connected by nerve fibers which communicate information. In our models, owing to an absence of high-resolution brain structural data, the Euclidean distance was used to estimate the information transmission distance between brain regions. The Euclidean distance was used as property in a recent study on the evolution model to simulate the formation of brain network, and the results are indeed satisfying [31], proving that the estimation of information transmission distance is feasible.

Brain regions represented by nodes in brain network contained the area of cerebrum, but not the cerebellum. Because the cerebellum receives information from cerebrum to control movement, we considered it outside the focal area. Accordingly, our brain network consists of cerebrum only and reflects the brain activities in the finger task.

## Conclusion

On the basis of a computational experiment, we have explored a probabilistic model to parsimoniously simulate the evolution process of brain networks in the acute period from pre-stroke to two weeks after stroke. The evolution model has been effectively applied to simulate the evolution of stroke-affected brain networks in our study. In cases where experimental data is limited, dynamic information was obtained by simulation. This paper provides a novel approach toward investigating mechanisms of brain changes under conditions of neural development disorder or brain injury. In the acute period, the action of distance penalization may be used to describe the general mechanism of brain network evolution. Our study, through the use of evolution model, may contribute to a more comprehensive simulation of brain networks to aid in functional evaluation of stroke patients and is broadly applicable to research of stroke recovery processes.
